# Uncertainty in Muscle–Tendon Parameters can Greatly Influence the Accuracy of Knee Contact Force Estimates of Musculoskeletal Models

**DOI:** 10.3389/fbioe.2022.808027

**Published:** 2022-06-03

**Authors:** Seyyed Hamed Hosseini Nasab, Colin R. Smith, Allan Maas, Alexandra Vollenweider, Jörn Dymke, Pascal Schütz, Philipp Damm, Adam Trepczynski, William R. Taylor

**Affiliations:** ^1^ Laboratory for Movement Biomechanics, ETH Zürich, Zürich, Switzerland; ^2^ Aesculap AG, Tuttlingen, Germany; ^3^ Department of Orthopaedic and Trauma Surgery, Ludwig Maximilians University Munich, Musculoskeletal University Center Munich (MUM), Campus Grosshadern, Munich, Germany; ^4^ Julius Wolff Institute, Berlin Institute of Health at Charité—Universitätsmedizin Berlin, Berlin, Germany

**Keywords:** probabilistic analysis, musculoskeletal modeling, uncertainty, muscle parameters, knee contact force

## Abstract

Understanding the sources of error is critical before models of the musculoskeletal system can be usefully translated. Using *in vivo* measured tibiofemoral forces, the impact of uncertainty in muscle–tendon parameters on the accuracy of knee contact force estimates of a generic musculoskeletal model was investigated following a probabilistic approach. Population variability was introduced to the routine musculoskeletal modeling framework by perturbing input parameters of the lower limb muscles around their baseline values. Using ground reaction force and skin marker trajectory data collected from six subjects performing body-weight squat, the knee contact force was calculated for the perturbed models. The combined impact of input uncertainties resulted in a considerable variation in the knee contact force estimates (up to 2.1 BW change in the predicted force), especially at larger knee flexion angles, hence explaining up to 70% of the simulation error. Although individual muscle groups exhibited different contributions to the overall error, variation in the maximum isometric force and pathway of the muscles showed the highest impacts on the model outcomes. Importantly, this study highlights parameters that should be personalized in order to achieve the best possible predictions when using generic musculoskeletal models for activities involving deep knee flexion.

## Introduction

Musculoskeletal models have been widely used to estimate *in vivo* loading conditions within the knee (Richards and Higginson, 2010; Worsley et al., 2011; Trepczynski et al., 2012; Gerus et al., 2013; Stylianou et al., 2013; Chen et al., 2016; Trepczynski et al., 2018; van Rossom et al., 2018; Imani Nejad et al., 2020). Outputs of musculoskeletal simulations can be used to predict postoperative functional outcomes of different surgeries (Barry et al., 2010; Chen et al., 2016), optimize rehabilitation protocols (Barry et al., 2010; Jamwal et al., 2020; Li et al., 2020), and enhance athletic performance (Heron, 2015; Langholz et al., 2016; Ataei et al., 2020; Seow et al., 2020). However, when musculoskeletal predictions of knee loads are compared against *in vivo* measurements, substantial errors are common (Lundberg et al., 2012; Valente et al., 2014; Charles et al., 2020; Koller et al., 2021), especially when generic models are used (e.g., errors of up to 150% for body-weight squat (Schellenberg et al., 2018; Imani Nejad et al., 2020)).

Generic musculoskeletal models are generally developed based on averaged anatomical data obtained from cadavers or living subjects (Delp et al., 1990; Arnold et al., 2010; Rajagopal et al., 2016). To represent a specific subject, certain model parameters are usually scaled based on the individual’s weight and skin marker locations in a static pose (Kainz et al., 2017; Imani Nejad et al., 2020; Ma et al., 2021). However, the scaled parameters possess inherent uncertainty due to marker placement inaccuracy, on top of considerable inter-subject variability due to skeletal morphology, for example, femoral anteversion angle (Kainz et al., 2021; Modenese et al., 2021) and muscle parameters, for example, tendon slack length (Winby et al., 2008; Heinen et al., 2016). As a result, the importance of using subject-specific muscle parameters to reduce uncertainty in modeling outcomes has been consistently emphasized in the literature (Barry et al., 2010; Gerus et al., 2013; Myers et al., 2015; Chen et al., 2016; Koller et al., 2021), but such parameters are generally difficult to measure. For example, to determine the exact muscle pathway, multiple MR images should be captured along the length of the muscle and preferably during different body poses (Schmid et al., 2009; Fernandez et al., 2016). Muscle force generation parameters also play an important role: the maximum isometric force (MIF) of muscles can be estimated during maximum voluntary contraction trials using either force or torque sensors (Be Groote et al., 2010; Ivanovic and Dopsaj, 2013; Kainz et al., 2018; Higa et al., 2019) or electromyography (EMG) data (Pizzolato et al., 2015; Hoang et al., 2019); however, these assessments are generally not sufficiently repeatable or sensitive to determine the MIF of a single muscle (Bolliger et al., 2008). It is also known that EMG signals measured during some dynamic activities can exceed the values measured during maximum voluntary contraction trials (Burden, 2010), thus the measured MIF may not accurately represent the real muscle strength. As a result, measurement and implementation of all subject-specific muscle parameters for all muscles modeled is extremely difficult, expensive, and consequently impracticable. Therefore, it is crucial to limit subject-specific measurements to the muscles and parameters that have a considerable impact on the modeling outputs.

A probabilistic analysis can provide important insights into the relative impact of different sources of uncertainty on the muscle and joint reaction force estimates of generic musculoskeletal models (Barry et al., 2010; Valente et al., 2014; Myers et al., 2015; Lamberto et al., 2017; Zuk et al., 2018). Using experimental gait data collected from a single subject as inputs to a generic musculoskeletal model, Myers et al. (2015) found that measurement inaccuracies including marker placement and skin movement artifact have a relatively small contribution toward the overall uncertainty in muscle force estimates for level walking (e.g., 20 N variation in estimated gastrocnemius force due to marker placement errors, compared to 80 N variation due to errors in muscle input parameters). Nevertheless, they found 1.7 times greater impact on muscle force outputs due to uncertainty in muscle parameters, particularly maximum isometric force and tendon slack length. Similar findings were reported by Navacchia et al. (2016), who used a global probabilistic modeling approach to assess uncertainty in knee contact forces (KCFs). Although these investigations provide an underlying understanding of how uncertainties propagate through different stages of musculoskeletal simulations, they generally focused on level walking with experimental data collected from a single subject and without reliable *in vivo* data for validation. As a result, to date, there is no comprehensive probabilistic analysis that provides a reference for guiding the selection of influential model parameters that need to be personalized, especially for activities involving deep knee flexion.

In this study, a probabilistic musculoskeletal modeling approach was exploited to quantify the impact of uncertainty in lower limb muscle parameters on KCF estimates of a generic musculoskeletal model used to simulate squat activity. We used skin marker trajectories, ground reaction forces (GRF), and *in vivo* measured tibiofemoral joint contact forces reported within the CAMS-Knee data sets (Taylor et al., 2017) to perform the simulation and validate the results. The findings of this study aim to provide an improved understanding of the influence of model uncertainty on musculoskeletal simulation outcomes, as well as highlight parameters that should be personalized in order to achieve the best possible predictions of knee contact force.

## Methods

Skin marker trajectories, ground reaction forces, and *in vivo* KCF data, measured in six subjects (74 ± 6 years, 89 ± 13 kg, and 172 ± 4 cm) with an instrumented knee implant (INNEX FIXUC, Zimmer, Switzerland) were obtained from the CAMS-Knee data sets (Taylor et al., 2017).

A generic musculoskeletal model (Rajagopal et al., 2016) developed within the OpenSim modeling environment (Delp et al., 1990) with 37 degrees of freedom (DoFs) and 80 muscle–tendon units actuating the lower limb was selected for this study. To obtain the so-called baseline models representing each subject’s anthropometry, the position of selected bone landmarks and anatomic joint centers (3D position of these landmarks are extracted from the segmented CT images and available within the CAMS-Knee data sets) were used to scale each bone in the lower limb. More specifically, the 3D distances between six bone landmarks on the pelvis (left and right anterior and left and right posterior iliac spine as well as the two hip joint centers) were calculated based on the data obtained from CT images and compared against the distance between corresponding landmarks on the generic bone to scale the pelvis in three dimensions. The distance between proximal and distal joint centers was used to scale the lengths of the tibiae and femora. An average scale factor was calculated based on the distance between the medial and lateral epicondyles as well as the distance between the medial and lateral malleoli to scale the width and thickness of each femur, tibia, and patella. The feet and upper limb segments were scaled based on skin marker positions in a static pose. Optimal fiber length and tendon slack length were scaled based on the standard methods built into the OpenSim Scale Tool, while maximum isometric forces of the muscles were taken directly from the generic model.

**FIGURE 1:**
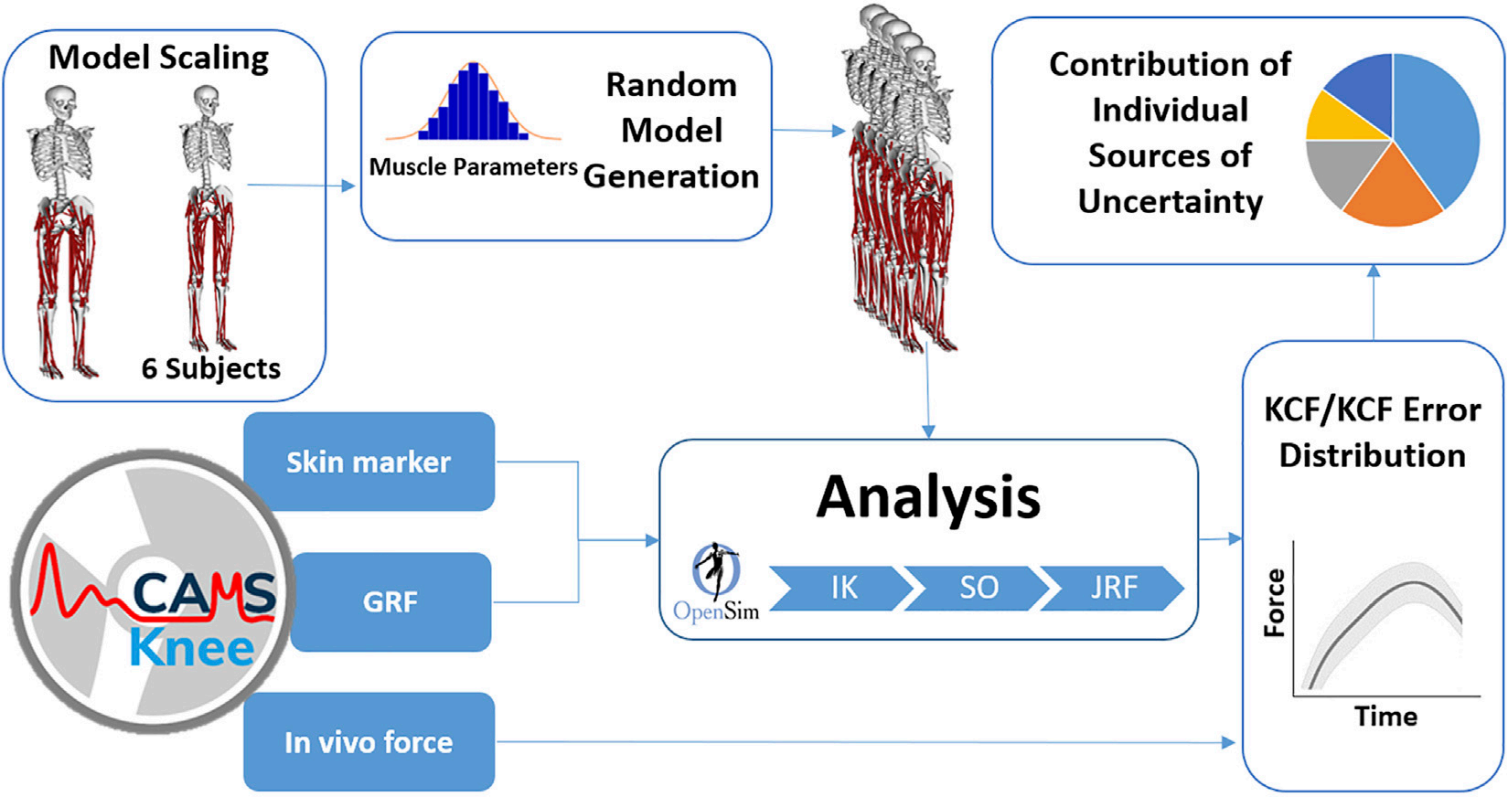
Probabilistic modeling flowchart. GRF, ground reaction force; IK, inverse kinematics; SO, static optimization; JRF, joint reaction force; KCF, knee contact force.

Muscles in the generic model were represented by an elastic tendon Hill-type muscle model (Millard2012EquilibriumMuscle (Millard et al., 2013)) with MIF, tendon slack length (TSL), and pennation angle (PEN) obtained from cadavers (Ward et al., 2009; Handsfield et al., 2014). The pathway of each muscle–tendon unit was specified using its origin and insertion points (OIPs), as well as via points (VIA) and/or wrapping objects, which were additionally used to represent the curvilinear path of the muscles. Using the OpenSim/MATLAB application programming interface, values for MIF, PEN, TSL, OIP, and VIA of seven major groups of the lower limb muscles (Table 1) were perturbed around their baseline values to perform a series of Monte Carlo (MC) simulations (Figure 1). Here, in addition to a general MC analysis where all muscles of the lower limb were perturbed simultaneously, individual MC analyses were performed to assess the relative contribution of the knee (flexors and extensors), ankle, and hip muscles toward the overall uncertainty in KCF estimates. In addition, the biarticular muscles were investigated separately to understand how much uncertainty propagates from neighboring joints to the knee.

**TABLE 1:** Skeletal muscle groups included in the probabilistic simulation.

Muscle group	Muscles in the musculoskeletal model
Lower limb muscles	Adductor brevis (addbrev), adductor longus (addlong), adductor magnus distal (addmagDist), adductor magnus ischial (addmagIsch), adductor magnus middle (addmagMid), adductor magnus proximal (addmagProx), biceps femoris long head (bflh), biceps femoris short head (bfsh), extensor digitorum longus (edl), extensor hallucis longus (ehl), flexor digitorum longus (fdl), flexor hallucis longus (fhl), lateral gastrocnemius (gaslat), medial gastrocnemius (gasmed), gluteus maximus (glmax, 3 bundles), gluteus medius (glmed, 3 bundles), gluteus minimus (glmin, 3 bundles), gracilis (grac), iliacus, peroneus brevis (perbrev), peroneus longus (perlong), piriformis (piri), psoas, rectus femoris (recfem), sartorius (sart), semimembranosus (semimem), semitendinosus (semiten), soleus, tensor fasciae latae (tfl), tibialis anterior (tibant), tibialis posterior (tibpost), vastus intermedius (vasint), vastus lateralis (vaslat), and vastus medialis (vasmed)
Knee extensors	Recfem, vasint, vaslat, and vasmed
Knee flexors	bflh, bfsh, gaslat, gasmed, grac, sart, semimem, and semiten
Hip muscles	addbrev, addlong, addmagDist, addmagIsch, addmagMid, addmagProx, glmax (3bundles), glmed (3 bundles), glmin (3 bundles), iliacus, piri, psoas, and tfl
Ankle muscles	edl, ehl, fdl, fhl, perbrev, perlong, soleus, tibant, and tibpost
Knee-hip biarticular muscles	bflh, grac, recfem, sart, semimem, and semiten
Knee-ankle biarticular muscles	gaslat and gasmed

The muscle–tendon model parameters were probabilistically represented as Gaussian distributions based on the inter-individual variability reported in the literature (Friederich and Brand, 1990; Fukunaga et al., 1996; Lloyd and Besier, 2003; Ward et al., 2009), Supplementary Table S1). In case different coefficients of variation were reported for the same parameter, the weighted average coefficient was used, where the weight was determined based on the number of studied subjects. If the parameter variability for a specific muscle was not available in the literature, the mean of the reported coefficients of variation for other muscles was used to sample random input parameters. Regarding lack of information about the range of variability in muscle pathway parameters, similar to a previous probabilistic study (Navacchia et al., 2016), 5 mm was used as the standard deviation for OIP and VIA distributions.

To assess the overall impact of uncertainty in muscle model parameters on predicted muscle and knee contact forces, we performed one general MC simulation with 2000 iterations for each subject and muscle group, where all the five input parameters of the included muscles were perturbed. In addition, to assess the isolated effect of each source of uncertainty, five individual MC simulations each with 500 iterations were performed where only a single muscle parameter was perturbed at a time. The number of iterations was chosen based on convergence at approximately 1,600 and 350 simulations during early model testing. Here, similar to previous probabilistic musculoskeletal modeling studies (Valente et al., 2014; Myers et al., 2015), convergence of the Monte Carlo simulations was ensured when adding 10% more iterations resulted in less than 1% change in the mean confidence bound of the outcome KCF. In order to ensure that convergence could be reasonably reached for all models within the analysis pipeline, higher values of 2000 and 500 iterations were chosen. During post-processing, all simulations were checked and confirmed that convergence had indeed been reached within these provided analysis conditions. As a result, the designed MC had a total number of 189,000 iterations.

For each subject, skin marker trajectory and GRF data of a single representative squat trial were input to the standard OpenSim tools. Inverse kinematics (IK), static optimization (SO), and joint reaction force (JRF) analysis tools were used to calculate muscle activation and total KCFs in the baseline as well as in the perturbed models by minimizing the sum of the squared muscle activations. To assess the influence of using more simplified muscle models, baseline model simulations were repeated without muscle–tendon force–length characteristics in the static optimization tool.

The measured EMG signals were processed within MATLAB (R2017b, MathWorks, United States) where the raw signal was bandpass filtered (fourth order Butterworth, lowpass 10 Hz, and highpass 300 Hz), offset corrected, rectified, and finally lowpass filtered using a moving average filter. The maximum activation value recorded across all trials of all activities performed for each subject was used to normalize the processed EMG signals. Regarding measurement errors/artefacts and uncertainty introduced by normalization technique, EMG signals were only used for qualitative validation of the muscle activation patterns estimated by the baseline models. In addition, the 5th–95th percentile range of the muscle activation outputs was used to understand how model parameter uncertainty influenced muscle redundancy solutions. The calculated 5th–95th percentile range of the KCF error distributions obtained from general MC was compared between different muscle groups to identify the muscle group with the highest contribution toward the overall KCF uncertainty. In addition, for each muscle group, the ratio between 5th–95th percentile range of individual and general MC outputs was calculated to assess the relative contribution of each muscle parameter to the overall uncertainty observed in the KCFs. Finally, perturbed models with the least root mean squared error (RMSE) between predicted and measured KCFs were selected to understand whether subject-specific measurement of all muscle parameters can guarantee fully accurate KCF estimates when squat activity is simulated using generic models.

## Results

**FIGURE 2:**
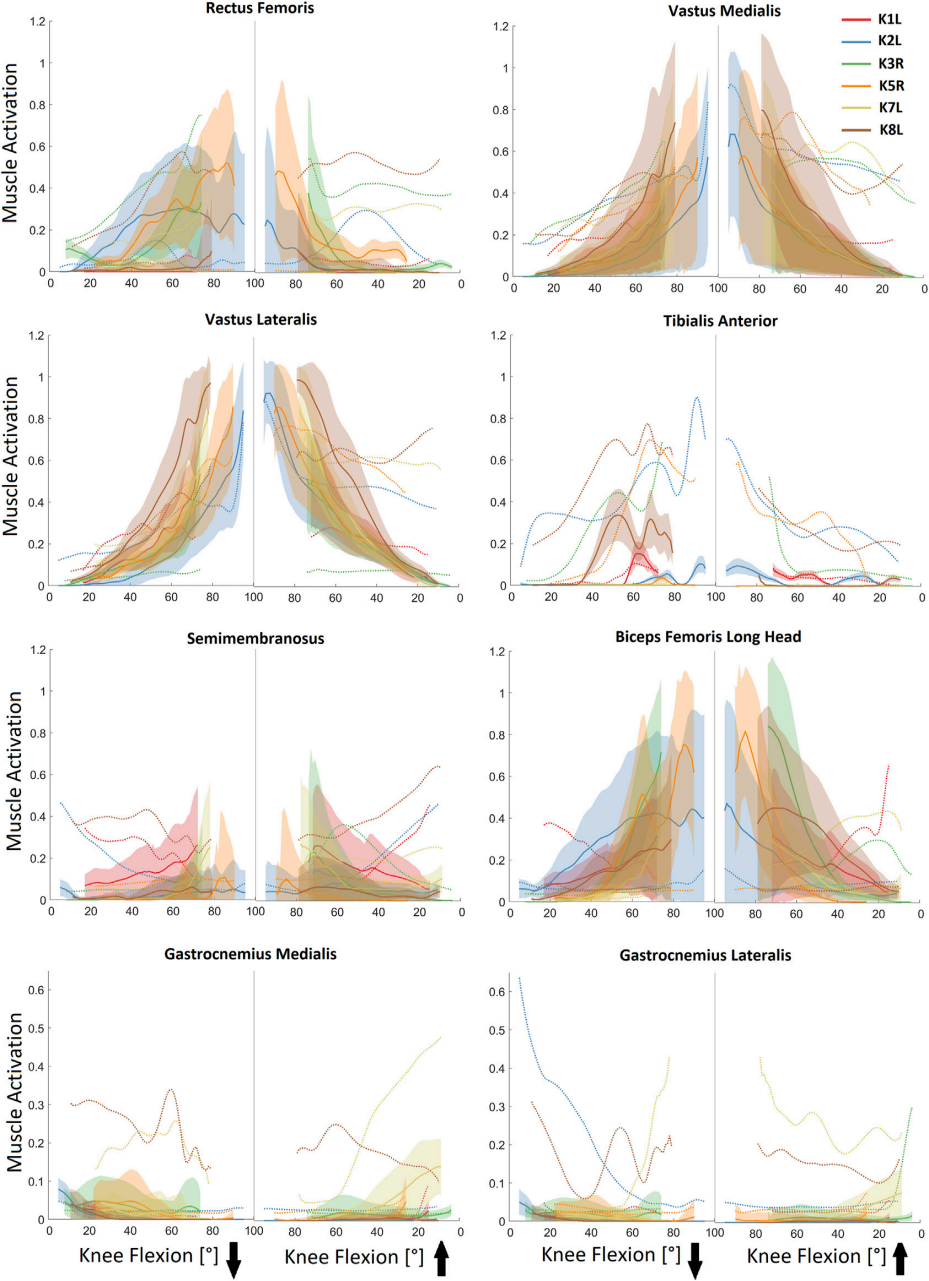
*In vivo* measured EMG (dotted lines) vs. predicted muscle activation levels for selected lower limb muscles during the studied squat trials. Solid lines represent average (baseline) activation patterns obtained from baseline models, whereas shaded areas display 5th–95th percentile range of the muscle activations obtained from general Monte Carlo simulations. The downward and upward arrows represent the descending and ascending phases of the squat activity.

Activation patterns of the knee extensor muscles predicted by the baseline models displayed similar trends to the measured EMG signals, especially during the descending phase of the squat (Figure 2). The flexion-dependent increase in EMG patterns of the rectus femoris and vastii was also present in the simulation outcomes. However, while the simulation results showed minimal activation of almost all muscles at the end of the squat cycle, the measured EMG signals indicated some residual co-contraction of the extensor and flexor muscles. The estimated muscle activation for the biceps femoris long head displayed a distinct peak at deep knee flexion, where the EMG signals showed minimal activation of this muscle. The models predicted very minimal activation of the gastrocnemii throughout the entire squat cycle, which was also observed in the EMG signals. However in some subjects, EMG sensors measured a relatively high activation in these muscles at the beginning or end of the activity cycle.

Simultaneous perturbation of the MIF, PEN, TSL, OIP, and VIA parameters of the lower limb muscles resulted in variations of up to 80% in the activation levels of the rectus femoris, vastus medialis, and biceps femoris long head, as well as up to 50% variation in activation of the vastus lateralis and semimembranosus (Figure 2). These variations were generally subject-specific and flexion-dependent. However, the impact of uncertainty in input parameters on the activation of tibialis anterior and gastrocnemii muscles was generally very small (Figure 2).

**FIGURE 3:**
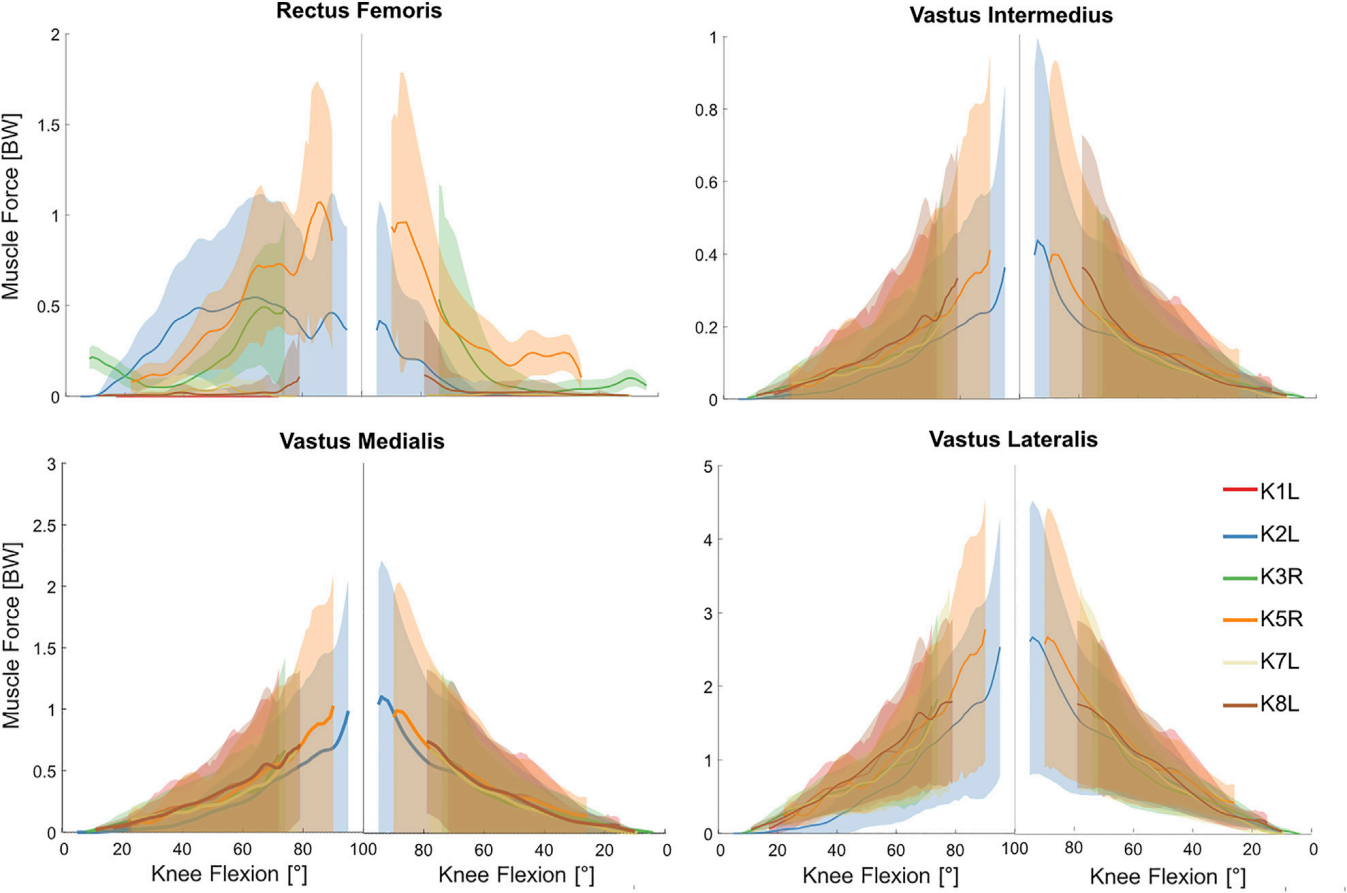
Estimated forces in the knee extensor muscles obtained from the baseline musculoskeletal models (solid lines) and 5th–95th percentile range of the general Monte Carlo simulation outputs (shaded area) for the studied squat trials.

**FIGURE 4:**
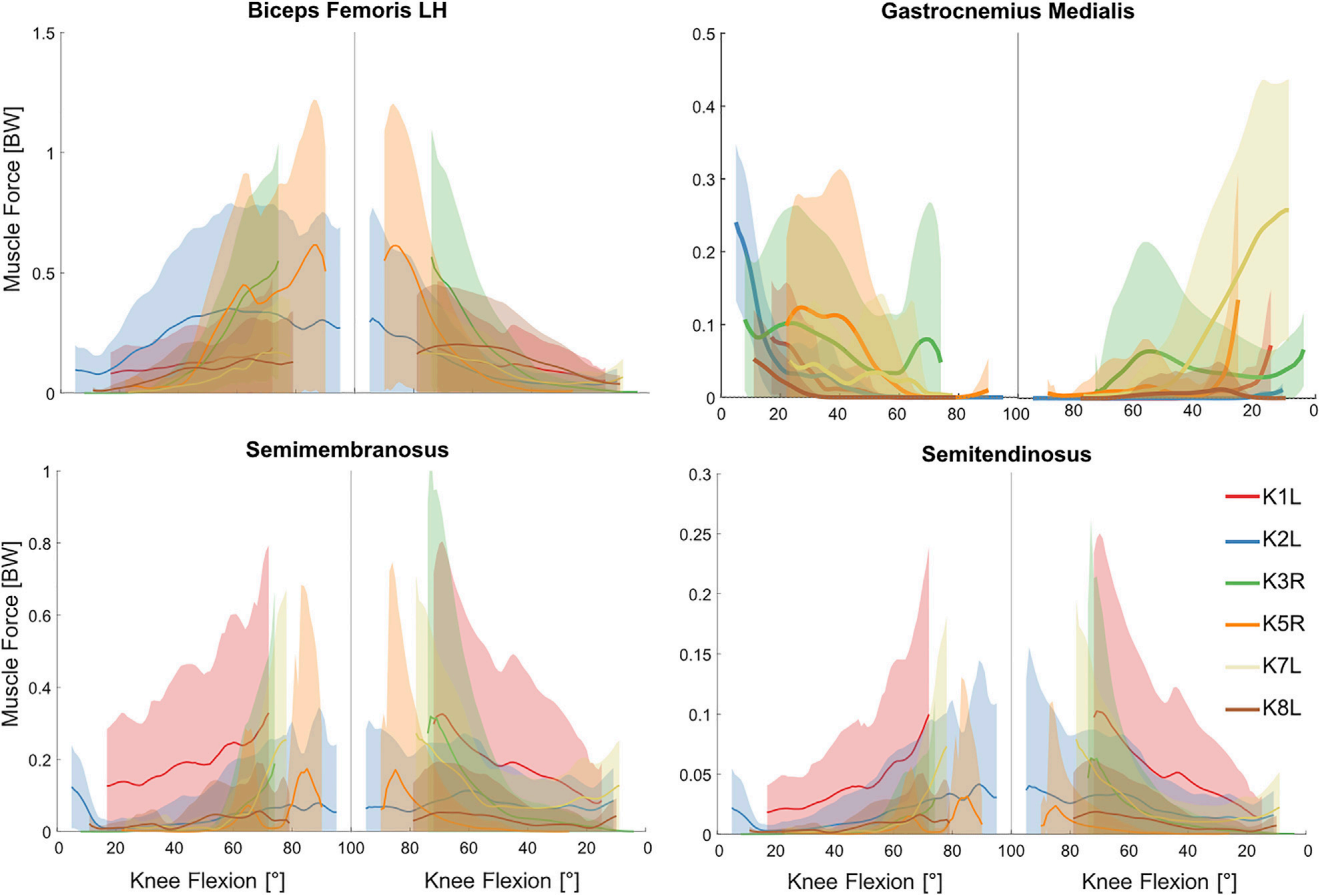
Estimated forces in the knee flexor muscles obtained from the baseline musculoskeletal models (solid lines) and 5th–95th percentile range of the general Monte Carlo simulation outputs (shaded area) for the studied squat trials.

As required to balance the external knee moment, predicted muscle forces from the baseline models generally showed the greatest forces in the knee extensor muscles (Figure 3). For example, vastus lateralis force reached 1.8–2.5 BW at the instance of deepest squat. However, antagonist forces were also predicted in the biarticular knee flexors to generate the necessary hip and ankle moments (Figure 4). Here, the greatest force in the knee flexors was about 0.6 BW generated by the biceps femoris long head. Interestingly, forces in the vastus muscles were clearly flexion-dependent, while other knee muscles showed subject-specific force patterns. During the flexion phase of squat, force in the gastrocnemius muscles exhibited a rapid decline, directly after knee flexion was initiated, followed by a gradual decrease with increasing knee flexion angle (Figure 4). During the extension phase, this pattern was reversed.

Simultaneous perturbation of the input parameters of all lower limb muscles resulted in considerable variations in the estimated muscle forces (Figures 3, 4 and Supplementary Figures S1, S2). These variations were generally subject-specific (e.g., 3.4 BW variation in vastus lateralis force for K2L compared with 2.2 BW for K8L, both values occurring at deepest squat) and more pronounced at larger knee flexion angles.

**FIGURE 5:**
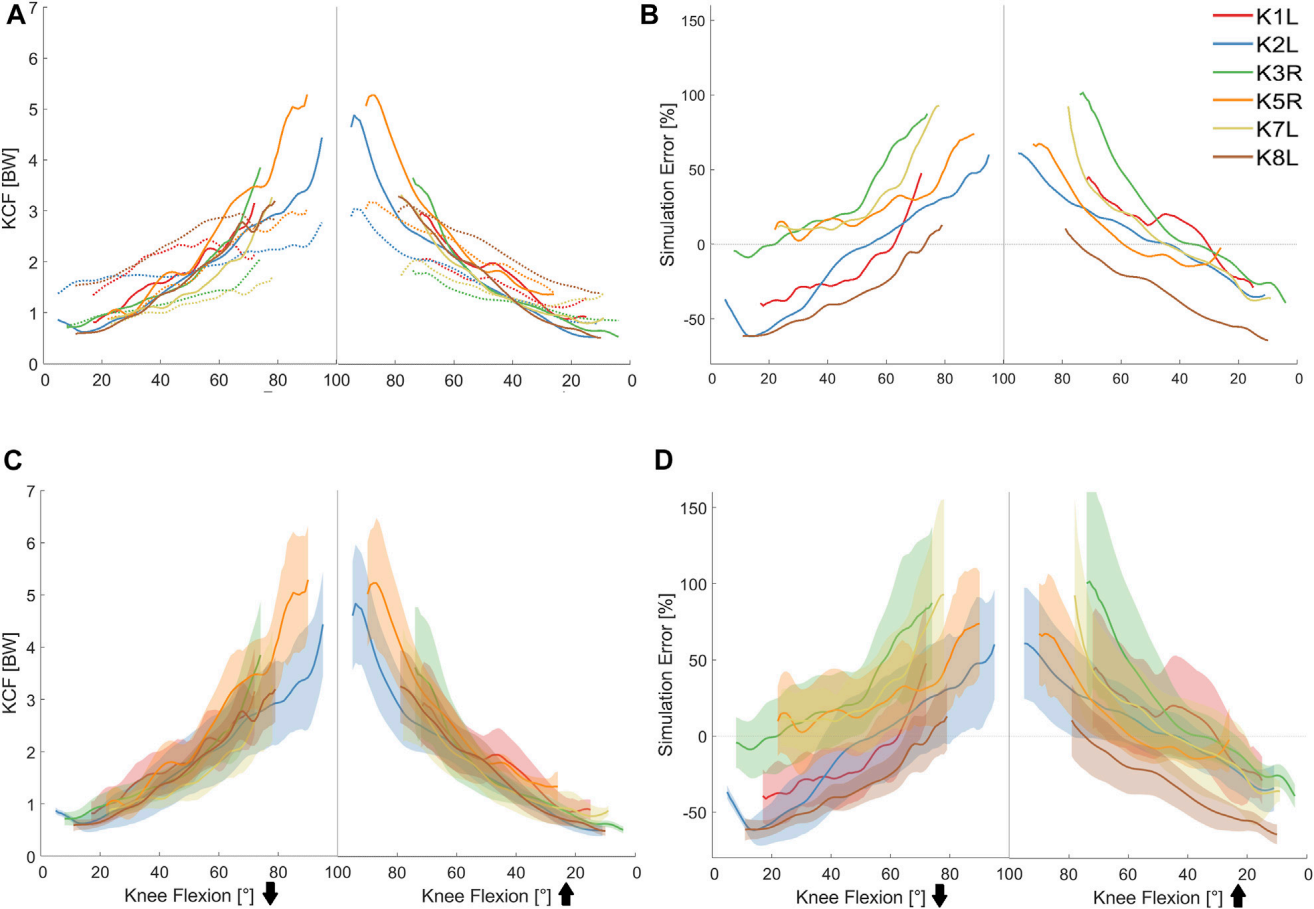
**(A)** *In vivo* measured (dotted lines) vs. predicted knee contact forces (solid lines) for the studied squat trials. **(B)** Simulation errors of the baseline musculoskeletal models. **(C)** Mean (solid lines) and 5th–95th percentile range (shaded area) of the knee contact force obtained from general Monte Carlo simulation outputs. **(D)** Mean (solid lines) and 5th–95th percentile range (shaded area) of the modeling error calculated from general Monte Carlo simulations.

Baseline simulations showed relatively large inter-subject variability in KCF estimates, with peak forces ranging from 3 to 5 times body weight. However, the KCFs showed a consistent flexion-dependent pattern across the studied subjects. Simulation error exhibited a similar flexion-dependent trend with the largest errors of up to 100% occurring at deepest squat when compared to the *in vivo* measured forces (Figure 5). Using simplified muscle models (no muscle force-length characteristics) resulted in a general increase in KCF estimates (up to 50% BW change in the KCF predictions of the baseline OpenSim models) and up to 50% change in the corresponding simulation errors (Supplementary Figure S3).

**FIGURE 6:**
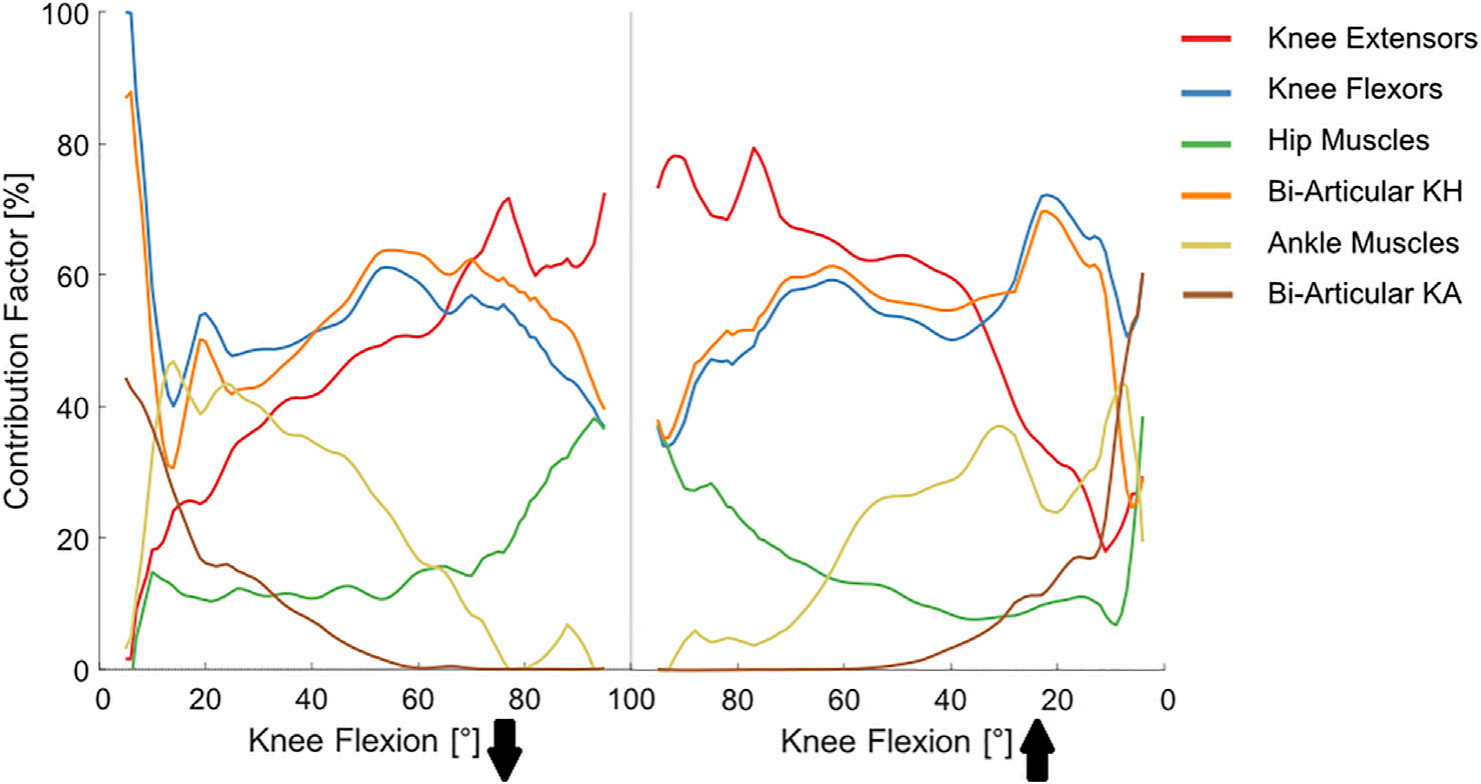
Contribution of different muscle groups toward the overall variability in KCF estimates. Solid lines represent average contributions obtained from the general Monte Carlo analysis with a total number of 72,000 joint reaction force simulations (2000 iterations per subject and muscle group). KH (Knee-Hip) and KA (Knee-Ankle).

The MC simulations revealed that uncertainty in the lower limb muscle parameters can result in considerable variation of the KCF, especially at larger knee flexion angles (e.g., 2.1 BW at 88° knee flexion for K5R). Similarly, the KCF simulation error was greatly influenced by the input uncertainty and showed large variation at deep knee flexion that could explain up to 70% of the simulation error (Figure 5). Here, the knee flexor muscles showed the highest contribution (50–100%) to the overall KCF uncertainty at small flexion angles, whereas knee extensors were the main contributors (70–80%) at larger flexion angles (Figure 6). Variation of the hip muscle parameters had a very small impact until around 50° knee flexion but became more influential thereafter. Ankle muscles had a considerable contribution toward the overall uncertainty when the knee was close to full extension; however, their influence declined with increasing knee flexion angle.

**FIGURE 7:**
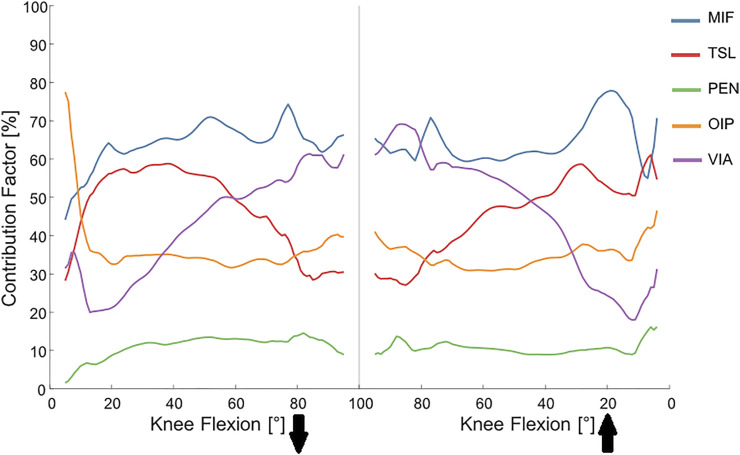
Contribution of different muscle parameters toward the overall variability in KCF estimates. Solid lines represent average contribution factors obtained from the general and individual MCs with a total number of 24,000 JRF simulations (4,000 iterations per subject). MIF, maximum isometric force; TSL, tendon slack length; PEN, pennation angle; OIP, origin and insertion point; VIA, VIA points.

Among the studied parameters, MIF and PEN were the most and least influential factors, explaining around 70% and 10% of the overall uncertainty (Figure 7; Table 2). Variability of the TSL had a considerable impact on the KCF estimates until around 50° knee flexion, but its influence declined thereafter. Interestingly, the contribution of the VIA parameter toward the overall KCF uncertainty was almost linearly dependent on the knee flexion angle, with around 60% contribution at 90° knee flexion. Here, perturbation of the VIA of the knee extensor muscles introduced up to 15 mm variation in the muscle moment arms (Supplementary Figure S4) that consequently resulted in around 1.2BW variation in the KCF estimates (also explaining up to 40% of the simulation error, Supplementary Figure S5). Similar to MIF and PEN, variability induced by OIP variation remained consistent over the entire range of knee flexion, explaining about 35% of the overall KCF variability.

**TABLE 2:** Contribution factor (in percent) of the input parameters of the studied muscle groups toward the overall variability in the knee contact force estimates at deepest knee flexion angle during squat.

	MIF	TSL	PEN	OIP	VIA
Lower limb muscles	66	30	9	40	61
Knee extensors	36	19	8	33	52
Knee flexors	31	22	4	18	26
Hip muscles	49	27	8	31	25
Knee-hip biarticular muscles	26	27	2	14	21
Ankle muscles	0	0	0	0	0
Knee-ankle biarticular muscles	0	0	0	0	0

Perturbed models with the lowest RMSE of the simulation results showed the greatest improvements in the KCF predictions (27% decrease in average RMSE, Supplementary Table S2 and Figure S6). However, these improvements were mostly highlighted at larger flexion angles where baseline model errors of up to 100% were decreased to less than 10% for models with optimized muscle parameters reported in Supplementary Table S3–S8. It should be noted, however, that even in these models with minimal KCF RMSE over the complete squat cycle, KCF predictions were not substantially better than those obtained from baseline models at the beginning and end of the cycle. Regarding the discrepancy between the estimated muscle activation and measured EMG signals (Figure 2), errors at the start and end of the squat cycle mainly originated from the inability of the static optimization method to account for the observed muscle co-contractions.

## Discussion

This study assessed the influence of muscle model parameter uncertainty on simulation predictions of a generic musculoskeletal model used to estimate KCFs in six instrumented TKA subjects performing a body-weight squat. The results indicate uncertainties in model parameters propagated through the simulation workflow, resulting in a significant impact on the muscle and knee joint contact force predictions. Simultaneous variation of the muscle force-length and geometric pathway parameters of the lower limb muscles resulted in more than 2 BW variations in the KCF and 70% variation in the modeling error. We also found that the contribution of different muscle groups and input parameters toward the overall uncertainty in the KCF outcome may vary over the range of knee flexion angle. These results indicate that, to reduce uncertainty in the predicted KCFs, subject-specific MIF and VIA of the lower limb muscles in generic models must be determined through measurement or calibration, especially when activities with larger flexion angles are considered.

Similar to the *in vivo* measured tibiofemoral forces, KCFs predicted by the baseline models indicated flexion-dependent patterns, with peaks occurring at the largest flexion angles during squat. While at small flexion angles, KCFs were both over- and under-predicted by the baseline models, at larger knee flexion angles, model estimates were substantially greater than the measured outputs. Other studies that used the CAMS-knee data sets have reported similar KCF errors obtained not only from the same generic model used in the current study but also when using a different generic model (Gait 2,392) (Schellenberg et al., 2018; Imani Nejad et al., 2020). In an another modeling study (Ding et al., 2016), when KCF estimates from squat simulation were compared against the *in vivo* data reported in the sixth Grand Challenge Competition (Fregly et al., 2012), errors of up to 100% BW were identified. van Rossom et al. (2018) used a six DoF knee model integrated in a full body musculoskeletal model to estimate KCFs in 15 young healthy subjects performing squats. They found an average maximum KCF of around 4 times BW, which is comparable with the results reported by Shelburne and Pandy (2002), who used a simpler musculoskeletal model. Despite using a different generic model, the results of our baseline model simulations concur well with those reported from these aforementioned studies. It is important to note, however, that the majority of predictions presented to date are generally higher than the tibiofemoral forces measured in living subjects performing squat (2.2-3BW, (Mundermann et al., 2008; Fregly et al., 2012; Trepczynski et al., 2012; Bergmann et al., 2014; Mizu-uchi et al., 2015; Catelli et al., 2019)).

We found varying contributions between the studied muscle groups toward the overall uncertainty in KCF estimates. Here, the knee flexor muscles played a greater role at lower flexion angles, while knee extensors became considerably more influential at larger flexion angles (Figure 6). We also found a slightly higher contribution of the knee extensor muscles during the extension phase of the squat compared with the flexion phase. These findings can be partially explained by the different activation patterns of the leg muscles during squat. Escamilla et al. (2001) measured EMG patterns of different muscle groups in 10 subjects and found that the peak quadriceps activity occurs at 80–90° knee flexion and that the activity is 25–50% greater in the knee extension phase. Conversely, knee flexor muscles are more active at smaller flexion angles during squat. In particular, at the beginning of the flexion phase, the biceps femoris, gastrocnemius, and semitendinosus all act together to initiate flexion of the knee (Robertson et al., 2008). Although knee flexor muscles remain less active after their initial burst (Wilk et al., 1996; Isear et al., 1997), biceps femoris long head, semimembranosus, and semitendinosus continue lengthening and thus apply a considerable passive force until around 60° flexion (Sinclair et al., 2017). From 60° to 90°, the hamstring muscles remain nearly isometric and minimally activated (Wilk et al., 1996; Isear et al., 1997; Sinclair et al., 2017). This can explain our results indicating a decline in contribution of knee flexors toward uncertainty in the KCF after 60° knee flexion. The increasing contribution of the hip muscles after around 50° knee flexion is likely due to the increases in hip moment magnitudes (in all 3 DoF) and increased inaccuracies in muscle pathway representations and resulting moment arms of these muscles.

MIF was found to be the most influential parameter on the resultant KCF throughout the entire range of knee flexion (Figure 7), which concurs with findings of previous probabilistic studies performed on level walking (Navacchia et al., 2016; Zuk et al., 2018). However, it should be mentioned that, due to the nature of SO, changing the MIF of all muscles surrounding the joint by the same amount (e.g., 30%) is unlikely to greatly alter the KCF estimates. Regarding the substantial inter-subject variability of MIF of the lower limb muscles (Friederich and Brand, 1990; Ward et al., 2009; Yucesoy et al., 2010; Bakenecker et al., 2020; Montefiori et al., 2020), using the baseline values from generic models may result in sizable KCF errors. Medical imaging techniques (e.g., MR and ultrasound imaging) and isokinetic dynamometers can be used to estimate subject-specific MIF of major muscle groups (Maganaris et al., 2001; Correa and Pandy, 2011; Ivanovic and Dopsaj, 2013; Kainz et al., 2018; Higa et al., 2019). Although, these methods have limited accuracies and are not applicable to all individual muscles, subject-specific determination of MIF inputs for major muscle groups seems to be able to considerably reduce uncertainty in KCF estimates of generic musculoskeletal models.

After MIF, VIA showed the largest impact on KCF predictions (Figure 7). Variation of the muscle VIA influences the muscle moment arm and thereby the recruitment strategy selected by the static optimization approach to balance the external joint moment. In the current study, variation in VIA was found to have a flexion-dependent contribution toward the overall KCF variability, with a maximum contribution of around 70% at deepest squat. Using a global probabilistic approach, Navacchia et al. (2016) found that the uncertainty in VIA can explain around 50% of the overall variability in the estimated KCFs for level walking. However, they did not provide details on how VIA contribution changes over the range of knee flexion. The large contribution of VIA uncertainty in our study is due to the high range of loaded knee flexion during squat, whereas the flexion-dependent pattern is likely to be associated with the decreased accuracy in muscle pathway representation in generic models. Musculoskeletal models have been mainly developed to assess walking tasks, and hence their muscle moment arms are validated for a small range of knee and hip flexion (Delp et al., 1990; Arnold et al., 2010; Rajagopal et al., 2016). Therefore, as the knee flexion angle increases, variation of the VIA points of the highly loaded muscles (mainly knee extensors) has a more highlighted effect on the KCF output. This is not limited to the knee muscles. For example, variation of the VIA of the hip muscles (mainly hip adductors) can change the entire force distribution between the hip muscles, which consequently affects the KCF due to the changes induced in biarticular muscle forces. Regarding the increasing contribution of VIA and extensor muscles with increasing knee flexion angle, the observed flexion-dependent errors in our baseline simulations most likely originate from inaccurate representation of the knee extensor muscle pathways in the scaled generic models. Our results, therefore, highlight the importance of subject-specific modeling of the muscle pathway geometry when activities with large knee flexions are investigated using generic musculoskeletal models. To reduce uncertainty in muscle moment arms, more accurate VIA pathways can be obtained from MRI images (Blemker et al., 2007; Scheys et al., 2008); however, implementation of these parameters in the model necessitates the use of simplified wrapping objects. As a result, even personalized models have an inherent uncertainty in their muscle geometry representation. Moreover, other sources of inter-subject variability such as bone geometry (e.g., femoral anteversion angle or tibial torsion) may also influence the muscle attachment points and pathway geometry, thereby impacting the predicted KCFs (Kainz et al., 2020; Modenese et al., 2021).

Our data suggest that parameters with low impacts can be directly taken from the generic model (or scaled based on subject’s anthropometry). The pennation angle of the muscle fibers (PEN) is a clear example of such a parameter, considering its relatively small impact on the KCF estimates (Figure 7, also reported by Navacchia et al. (2016)). For the studied squat cycles, TSL had a considerable impact on KCF variability, especially between 20° and 50° knee flexion. The large sensitivity of predicted KCFs to uncertainty in TSL has been also reported in previous studies on level walking (Myers et al., 2015; Carbone et al., 2016; Navacchia et al., 2016). However, plausibly due to the very large ±20% range of variation of the TSL (compared to 2–9% used in the current study), Carbone and co-workers found TSL to be more influential than MIF in prediction of the muscle forces. Regarding technical challenges in subject-specific measurement of TSL in living subjects, the baseline values from the generic models are either directly used or adjusted such that the differences between experimental and model-based joint moments is minimized (Van Campen et al., 2014; Heinen et al., 2019). It is also possible to ignore the force-length characteristics of the muscle-tendon units in the static optimization procedure. This approach excludes the role of the tendon slack length and optimal fiber length, while the maximum isometric force and muscle pathway geometry (which determines the muscle moment arm) remain influential. In the current study ignoring muscle force–length relationships did not play a dominant role in the knee contact force estimates; however, it did result in a general increase in KCF estimates and changes of up to 50% in simulation error (Supplementary Figure S3). Similar results were reported when muscle models were simplified during KCF estimation in level walking simulations (Modenese et al., 2018). Therefore, inclusion of force-length characteristics of the muscles in static optimization seems to be necessary in order to reduce uncertainty in modeling outcomes. Hence, a probabilistic modeling approach, as used in the current study, can provide an estimate of the range of possible errors that might originate from TSL uncertainty. Nevertheless, a complete understanding of the real influence of PEN and TSL on the model outcomes is limited by the 1D representation of the muscles in the selected musculoskeletal model. Therefore, volumetric modeling of muscles (Murai et al., 2016; Modenese and Kohout, 2020) may better clarify interaction between uncertainty in muscle parameters and variation in the joint contact force estimates, but such modeling approaches are extremely expensive and technically challenging.

Our results indicate that uncertainty in muscle parameters cannot completely explain KCF errors when generic models are used to simulate activities involving high knee joint flexion (Supplementary Table S2 and Figure S6). As a result, considerable errors, especially at small flexion angles, can still be expected even with subject-specific muscle parameters. A comparison between the predicted muscle activation and measured EMG signals (Figure 2) confirms the inability of utilized static optimization technique to account for co-contraction of the flexor and extensor muscles at the beginning and end of the squat cycle. Here, it is entirely plausible that subjects change neuromuscular control from stability and balance during standing to a more economical/preservation mechanism to avoid muscle overloading and/or damage during higher loading scenarios. Such control programs might nicely be accounted for through adapting the cost-function within the SO to account for, for example, muscle co-contractions. Thresholds or optimal approaches to implement and validate such novel methods would, however, need to be fully verified in future investigations.

Several limitations need to be considered when interpreting the results of this study. First, we chose the generic model developed by Rajagopal et al. (2016) because it has been widely used to assess KCFs during different activities. It is likely that uncertainty in input parameters may propagate differently into the outcomes of other generic models with different numbers of muscles and joint DoFs (Cleather and Bull, 2011; Kainz et al., 2016). Moreover, while CAMS-Knee data sets provide access to the fluoroscopically measured tibiofemoral implant kinematics, lack of subject-specific patellofemoral kinematics and bone geometries precluded prescription of the tibiofemoral kinematics within the IK process. As a result, similar to many other studies in the field, the model joints were driven by skin marker trajectories, and their kinematics may, therefore, differ from the subject-specific joint movements. In addition, while a large number of factors may affect the accuracy of musculoskeletal modeling knee contact force predictions, we only perturbed the muscle parameters in the selected generic musculoskeletal model. Although inclusion of other sources of uncertainty (e.g., skin marker location and body segment parameters) may also change the predicted results, previous probabilistic simulations reported no major influence of such factors (Valente et al., 2014; Myers et al., 2015; Navacchia et al., 2016). It is also important to mention that our probabilistic approach did not account for inter-subject variability of bone geometry. Such variations can impact the 3D pathways of the muscles and thereby the joint contact force estimation (Kainz et al., 2020; Modenese et al., 2021). More importantly, we only used SO to solve the muscle redundancy problem through minimizing the sum of the squared muscle activation levels. It is known that different optimization approaches may lead to different modeling outcomes (Li et al., 1998; Mokhtarzadeh et al., 2014). In addition, the unknown level of antagonistic muscle co-contraction (Trepczynski et al., 2018) and inability of the routine muscle optimization approaches to accurately predict such contributions (Mokhtarzadeh et al., 2014) may also hinder a clear understanding of uncertainty propagation in musculoskeletal modeling. Moreover, the assumed distributions for the uncertain muscle parameters also influence the variability in the predicted KCF, and since the range of variation in input parameters for some muscles was missing in the literature, the average of the reported values for other muscles was used to generate random inputs for the MC simulations. Finally, we assessed KCFs during squat cycles with a limited range of flexion performed by only six TKA subjects. Uncertainty propagation through the modeling framework may be different at larger knee flexion angles during squats performed by young healthy population.

## Conclusion

Personalization of musculoskeletal models remains technically and financially challenging. This study provides a more complete understanding of the impact of uncertainty in the input parameters on the estimated KCFs and can help to identify the most influential parameters that need to be accurately measured and implemented. The results suggest that attention should be paid to the subject-specific measurement of MIF and VIA in order to ensure errors in musculoskeletal modeling remain minimal. In addition, adaptive muscle optimization techniques that enable accurate prediction of the muscle co-contractions at the beginning and end of the squat cycle might well present improvements in KCF prediction deficits revealed in this study.

## Data Availability

Requests to access the data sets used in this study should be directed to: https://cams-knee.orthoload.com/data/data-download.
