# Erratum: Uncertainty in muscle-tendon parameters can greatly influence the accuracy of knee contact force estimates of musculoskeletal models

**DOI:** 10.3389/fbioe.2023.1182877

**Published:** 2023-03-16

**Authors:** Frontiers Production Office

**Affiliations:** Frontiers Media SA, Lausanne, Switzerland

**Keywords:** probabilistic analysis, musculoskeletal modeling, uncertainty, muscle parameters, knee contact force

An omission to the **Funding** section of the original article was made in error. The following sentence has been added: “Open access funding provided by ETH Zurich”

The publisher apologizes for this mistake. The original version of this article has been updated.

