# Supplementary material for: Uncertainty in Muscle–Tendon Parameters can Greatly Influence the Accuracy of Knee Contact Force Estimates of Musculoskeletal Models
Source: Front Bioeng Biotechnol. 2022 Jun 3;10:808027. doi: 10.3389/fbioe.2022.808027 (PMC9204520; doi:10.3389/fbioe.2022.808027)
Supplement: Supplementary file 1 [file DataSheet1.PDF]

Supplementary Material

Table S1: *Coefficient of variability (CoV) for the lower limb muscle parameters*

Muscle	MIF-CoV	Ref.	TSL-CoV	Ref.	PEN-CoV	Ref.
**bflh**	0.425	[1]	0.047	[2]	0.474	[1]
**bfsh**	0.333	[1]	0.067	[2]	0.293	[1]
**gaslat**	0.268	[1, 3, 4]	0.043	[2]	0.258	[1]
**gasmed**	0.207	[1, 3, 4]	0.016	[2]	0.444	[1]
**grac**	0.345	[1, 3]	0.010	[2]	0.305	[1]
**recfem**	0.330	[1, 3]	0.015	[2]	0.252	[1]
**sart**	0.375	[1, 3]	0.093	[2]	1.385	[1]
**semimem**	0.531	[1, 3]	0.073	[2]	0.225	[1]
**semiten**	0.423	[1, 3]	0.054	[2]	0.380	[1]
**vasint**	0.350	[1, 3]	0.022	[2]	1.000	[1]
**vaslat**	0.404	[1, 3]	0.033	[2]	0.370	[1]
**vasmed**	0.308	[1, 3]	0.044	[2]	0.233	[1]
**addbrev**	0.370	[1, 3]	0.042	[2]	0.508	[1]
**addlong**	0.336	[1, 3]	0.042	[2]	0.479	[1]
**addmagDist**	0.353	[1, 3]	0.042	[2]	0.173	[1]
**addmagIsch**	0.353	[1, 3]	0.042	[2]	0.173	[1]
**addmagMid**	0.353	[1, 3]	0.042	[2]	0.173	[1]
**addmagProx**	0.353	[1, 3]	0.042	[2]	0.173	[1]
**edl**	0.574	[1, 3, 4]	0.042	[2]	0.259	[1]
**ehl**	0.499	[1, 3]	0.042	[2]	0.234	[1]
**fdl**	0.433	[1, 3]	0.042	[2]	0.346	[1]
**fhl**	0.378	[1, 3]	0.042	[2]	0.272	[1]
**glmax**	0.263	[1]	0.042	[2]	1.20	[1]
**glmed**	0.426	[1]	0.042	[2]	0.844	[1]
**glmin**	0.353	[1]	0.042	[2]	0.173	[1]
**iliacus**	0.343	[1]	0.042	[2]	0.371	[1]
**perbrev**	0.386	[1, 3]	0.042	[2]	0.261	[1]
**perlong**	0.324	[1, 3]	0.042	[2]	0.362	[1]
**piri**	0.353	[1]	0.042	[2]	0.173	[1]
**psoas**	0.299	[1]	0.042	[2]	0.302	[1]
**soleus**	0.215	[1, 3, 4]	0.042	[2]	0.357	[1]
**tfl**	0.353	[1, 3, 4]	0.042	[2]	0.173	[1]
**tibant**	0.230	[1, 3, 4]	0.042	[2]	0.323	[1]
**tibpost**	0.379	[1, 3]	0.042	[2]	0.300	[1]

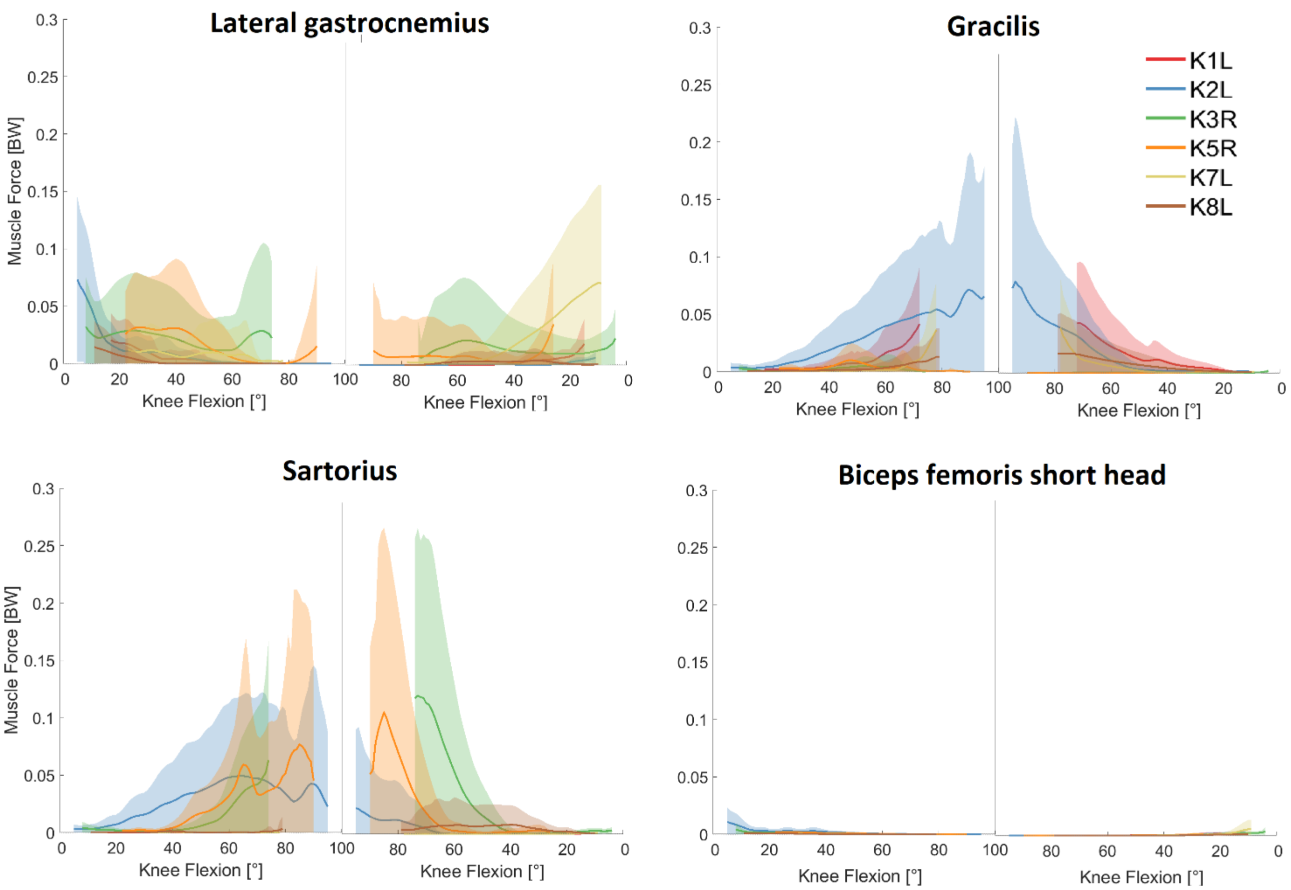

Figure S1: *Estimated force in lateral gastrocnemius, gracilis, sartorius, and biceps femoris short head obtained from the baseline musculoskeletal models (solid lines) and 5th-95th percentile range of the general Monte-Carlo simulation outputs (shaded area) for the studied squat trials.*

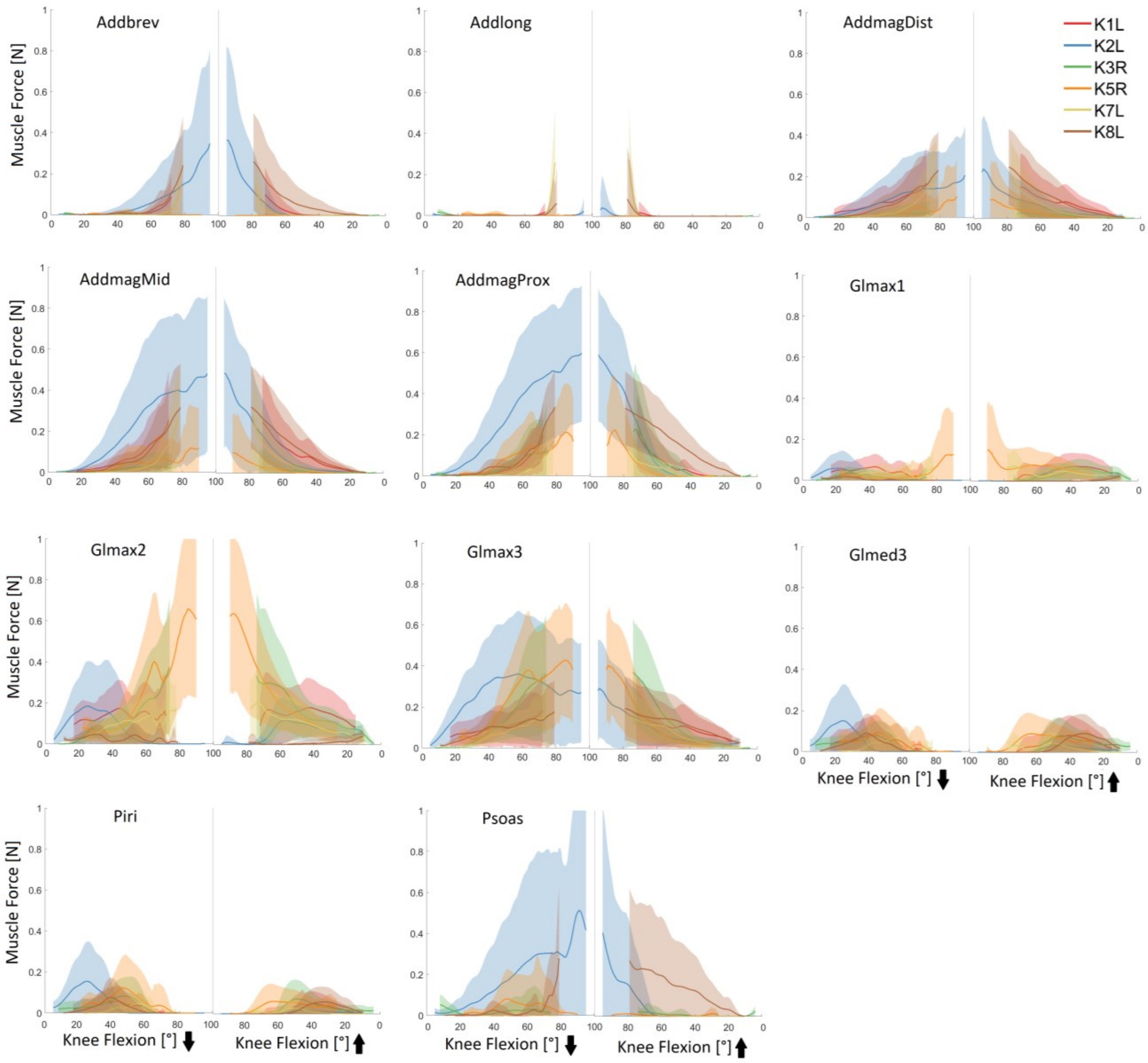

Figure S2: *Estimated force in the hip muscles obtained from the baseline musculoskeletal models (solid lines) and 5th-95th percentile range of the general Monte-Carlo simulation outputs (shaded area) for the studied squat trials.*

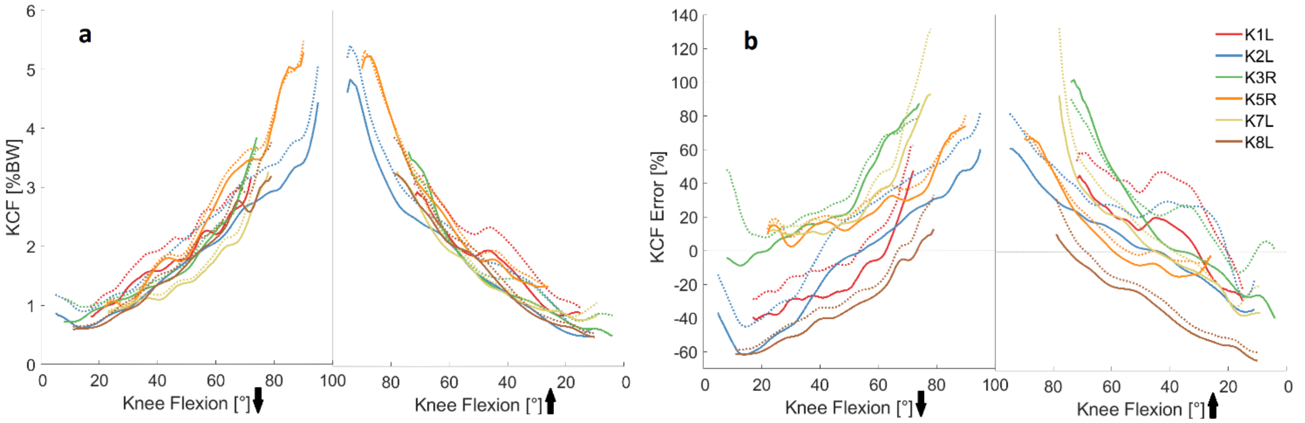

Figure S3: *Knee contact force estimation (a) and the corresponding simulation error (b) of the baseline musculoskeletal models with (solid lines) and without (dashed lines) accounting for the muscle force-length characteristics.*

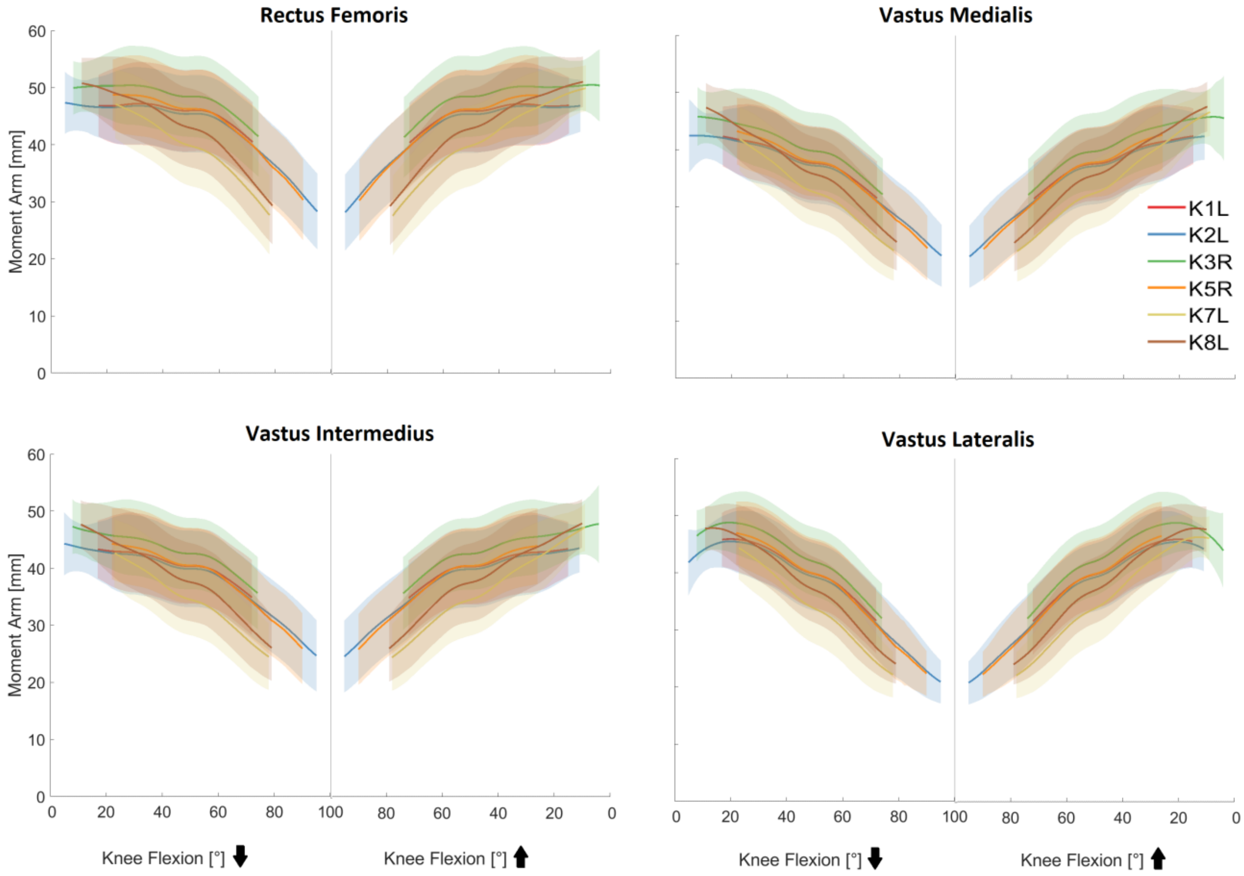

Figure S4: *Moment arms of the quadriceps in the baseline models (solid lines) and 5th-95th percentile range (shaded area) of the moment arm variation resulted from perturbation of muscle via points. Results obtained from a total number of 3000 random models (500 models per subject).*

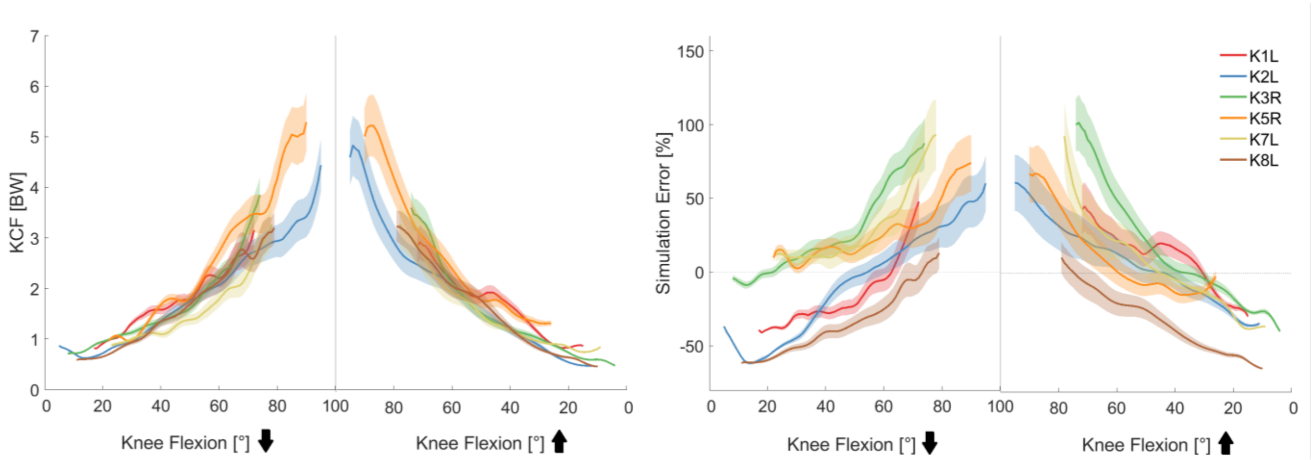

Figure S5: *Mean (solid lines) and 5th-95th percentile range (shaded area) of the knee contact force estimation (left) and the corresponding simulation error (right) resulted from perturbation of via points of the knee extensor muscles (MC with a total number of 3000 JRF simulations, 500 iterations per subject).*

Table S2: *Improvements in the root mean squared error of the knee contact force estimates of the musculoskeletal models with (Opt_RMSE [%]) and without (Gen_RMSE [%]) optimized inputs for the lower limb muscle parameters*

**Subject**	**Gen_RMSE [%]**	**Opt_RMSE [%]**
**K1L**	27	18
**K2L**	38	21
**K3R**	69	13
**K5R**	40	14
**K7L**	54	12
**K8L**	34	27
**Average**	44	17

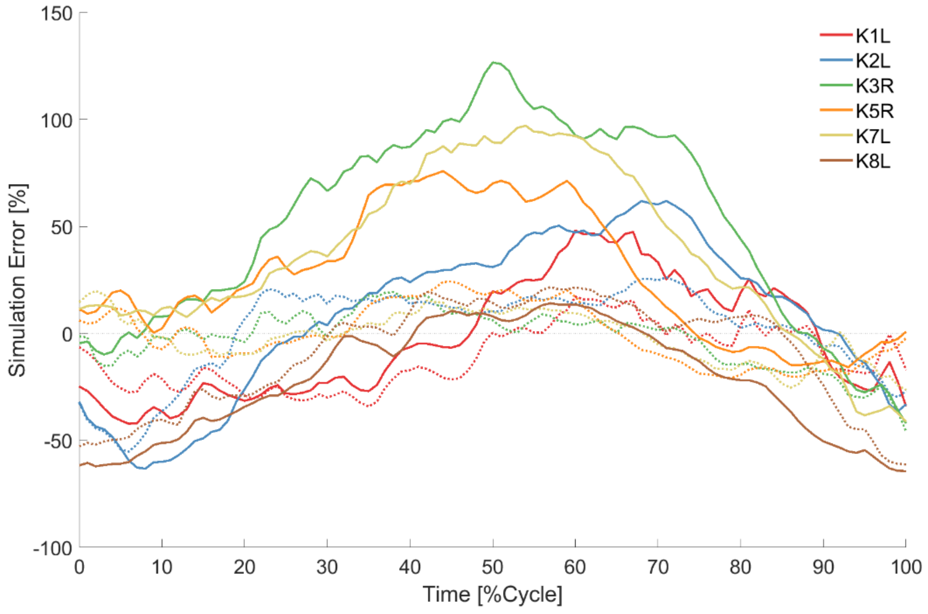

Figure S6: *Simulation error of the knee contact force estimates of the baseline models (solid lines) as well as errors of the perturbed models with the optimized muscle parameters that resulted in the lowest mean squared errors (dotted lines).*

Table S3: *Optimized inputs for the lower limb muscle parameters of the K1L subject*

**Muscle**	**MIF [N]**	**TSL [mm]**	**PEN [°]**
**bflh**	2077.66	313.32	15.7
**bfsh**	493.12	102.21	13.17
**gaslat**	1045.27	381.23	13.55
**gasmed**	4246.79	418.04	15.72
**grac**	207.22	157.47	7.98
**recfem**	2102.38	429.22	16.04
**sart**	394.52	133.51	1.28
**semimem**	3438.72	292.76	14.47
**semiten**	261.16	234.4	10.61
**vasint**	1800.94	198.11	1.9
**vaslat**	5478.04	214.25	17
**vasmed**	2082.8	202.08	26.58
**addbrev**	654.72	35.64	12.22
**addlong**	564	132.15	7.75
**addmagDist**	685.73	80.85	7.44
**addmagIsch**	608.88	197.76	9.41
**addmagMid**	843.6	44.51	14.61
**addmagProx**	345.17	45.03	17.43
**edl**	503.97	373.44	13.37
**ehl**	154.6	354.97	10.18
**fdl**	278.5	399.97	14.74
**fhl**	1008.56	367.52	10.04
**glmax1**	1184.72	54.66	7.12
**glmax2**	1218.51	76.77	22.56
**glmax3**	726.82	77.94	41.21
**glmed1**	1767.84	65.51	8.12
**glmed2**	505.39	82.07	10.84
**glmed3**	957.01	57.38	19.79
**glmin1**	406.31	19.9	8.74
**glmin2**	330.37	31	0
**glmin3**	486.61	57.14	0.89
**iliacus**	1064.83	115.65	22.38
**perbrev**	541.31	145.27	9.07
**perlong**	1757.03	335.11	14.14
**piri**	909.1	138.82	9.49
**psoas**	889.94	109.95	13.51
**soleus**	5526.55	310.18	24.08
**tfl**	301.4	447.21	2.94
**tibant**	1316.62	269.31	15.74
**tibpost**	2375.41	292.07	19.59

Table S4: *Optimized inputs for the lower limb muscle parameters of the K2L subject*

Muscle	MIF [N]	TSL [mm]	PEN [°]
**bflh**	2007.23	313.54	13.59
**bfsh**	457.87	98.78	17.6
**gaslat**	1344.11	389.26	15.15
**gasmed**	3074.93	411.39	9.02
**grac**	323.77	159.61	9.14
**recfem**	3384.01	439.48	12.64
**sart**	138.3	111.23	2.7
**semimem**	2078.47	314.09	11.26
**semiten**	477.31	219.37	14.55
**vasint**	1976.11	197.87	2.66
**vaslat**	7629.42	212.09	14.89
**vasmed**	2423.05	188.93	16.45
**addbrev**	198.4	35.62	8.72
**addlong**	977.07	123.21	15.38
**addmagDist**	889.54	73.71	12.29
**addmagIsch**	530.6	196.77	11.5
**addmagMid**	793.02	43.15	12.23
**addmagProx**	1000.17	43.14	17.75
**edl**	428.6	407.82	12.68
**ehl**	115.17	339.21	14.14
**fdl**	407.89	396.41	12.27
**fhl**	772.89	375.31	13.91
**glmax1**	1166.74	57.79	38.1
**glmax2**	1762.65	82.05	24.63
**glmax3**	646.75	78.75	6.67
**glmed1**	1221.99	70.25	15.35
**glmed2**	542.69	76.06	9.87
**glmed3**	134.86	57.61	35.63
**glmin1**	487.84	18.36	9.83
**glmin2**	139.77	29.02	0
**glmin3**	486.55	53.75	1.18
**iliacus**	1248.76	119.75	20.39
**perbrev**	196.84	143.33	7.01
**perlong**	753.62	349.19	8.4
**piri**	1012.24	135.29	10.31
**psoas**	1680	120.83	12.4
**soleus**	7281.97	298.12	30.6
**tfl**	669.72	450.88	3.85
**tibant**	1068.83	232.41	7.84
**tibpost**	1874.88	289.01	15.67

Table S5: *Optimized inputs for the lower limb muscle parameters of the K3R subject*

Muscle	MIF [N]	TSL [mm]	PEN [°]
**bflh**	411.07	307.76	17.12
**bfsh**	440.16	110.3	15.8
**gaslat**	2277.96	428.03	15.71
**gasmed**	2389.02	435.45	11.84
**grac**	424.64	170.47	5.06
**recfem**	2821.81	479.41	12.52
**sart**	282.18	120.21	2.7
**semimem**	1645.15	320.93	15.33
**semiten**	324.72	231.74	17.68
**vasint**	2480.86	216.82	0.83
**vaslat**	4303.4	231.59	8.64
**vasmed**	2408.09	196.61	20.11
**addbrev**	588.2	38.58	9.72
**addlong**	971.37	128.17	9.75
**addmagDist**	574.25	79.31	10.59
**addmagIsch**	701.31	205.67	8.23
**addmagMid**	940.83	45.66	14.11
**addmagProx**	610.9	44.03	13.03
**edl**	421.12	402.68	11.66
**ehl**	206.12	357.78	8.11
**fdl**	177.33	430.08	12.08
**fhl**	1047.71	399.08	17.85
**glmax1**	572.25	60.95	23.44
**glmax2**	1247.6	85.49	3.97
**glmax3**	1133.23	82.85	18.79
**glmed1**	1987.76	65.61	36.18
**glmed2**	691.26	79.67	18.77
**glmed3**	949.65	55.08	15.17
**glmin1**	122	19.3	9.21
**glmin2**	485.67	31.44	0
**glmin3**	615.52	61.9	0.88
**iliacus**	519.02	114.01	14
**perbrev**	383.17	154.38	10.55
**perlong**	1124.9	352.92	8.7
**piri**	1286.33	145.37	7.59
**psoas**	803.08	127.62	13.52
**soleus**	5485.77	310.69	31.04
**tfl**	594.9	490.32	2.96
**tibant**	1108.33	268.48	13.43
**tibpost**	2465.35	289.27	7.15

Table S6: *Optimized inputs for the lower limb muscle parameters of the K5R subject*

Muscle	MIF [N]	TSL [mm]	PEN [°]
**bflh**	277.42	336.75	13.57
**bfsh**	709.8	103.59	7.83
**gaslat**	1713.56	381.69	16.27
**gasmed**	3323.84	397.83	12.58
**grac**	241.89	174.1	9.35
**recfem**	2670.17	460.32	12.38
**sart**	202.47	131.69	0.42
**semimem**	654.9	331.73	15.86
**semiten**	249.9	251.62	16.93
**vasint**	1677.34	208.58	4.19
**vaslat**	7271.82	235.76	7.53
**vasmed**	2086.17	196.07	18.75
**addbrev**	589.76	40.18	9.46
**addlong**	1031.5	132.86	4.82
**addmagDist**	667.64	89.28	13.51
**addmagIsch**	707.11	208.69	7.15
**addmagMid**	762.35	47.98	9.7
**addmagProx**	443.21	46.79	18.59
**edl**	1048.65	406.12	7.82
**ehl**	255.03	350.02	13.62
**fdl**	132.52	421.92	9.68
**fhl**	518.27	391.59	20.34
**glmax1**	1106.99	60.03	13.62
**glmax2**	1331.09	77.14	36.08
**glmax3**	1114.22	81.98	14.29
**glmed1**	1635.31	67.17	16.65
**glmed2**	984.28	75.74	4.03
**glmed3**	142.21	57.03	14.7
**glmin1**	568.38	19.24	9.65
**glmin2**	610.22	31.54	0
**glmin3**	368.29	58.41	0.9
**iliacus**	893.29	116.35	13.42
**perbrev**	145.61	162.77	13.14
**perlong**	1007.82	339.21	20.76
**piri**	1001.75	129.13	8.79
**psoas**	1705.53	114.69	14.97
**soleus**	6391.27	301.98	16.74
**tfl**	592.79	468.7	2.75
**tibant**	1060.22	246.04	12.8
**tibpost**	1702.48	295.35	14.16

Table S7: *Optimized inputs for the lower limb muscle parameters of the K7L subject*

Muscle	MIF [N]	TSL [mm]	PEN [°]
**bflh**	680.67	312.82	13.4
**bfsh**	183.4	111.93	14.69
**gaslat**	782.71	392.44	15.13
**gasmed**	1714.78	429.82	10.94
**grac**	125.36	168.17	9.67
**recfem**	1226.25	439.93	16.44
**sart**	143.77	127.17	2.72
**semimem**	1043.17	305.41	13.71
**semiten**	275.61	244.88	7.79
**vasint**	1193.15	200.1	4.81
**vaslat**	2534.69	214.53	13.13
**vasmed**	757.26	190.68	20.08
**addbrev**	433.47	37.32	6.19
**addlong**	550.4	140.59	7.4
**addmagDist**	263.51	83.97	9.82
**addmagIsch**	214.18	190.34	8.54
**addmagMid**	173.19	50.85	9.23
**addmagProx**	467.27	51.32	14.47
**edl**	472.7	392.81	11.03
**ehl**	117.55	355.52	9.29
**fdl**	119.93	418.5	5.93
**fhl**	319.31	389.85	10.11
**glmax1**	521.04	53.37	27.64
**glmax2**	553.37	73.34	19.67
**glmax3**	437.24	75.91	35.11
**glmed1**	88.72	57.49	27.61
**glmed2**	442.6	70.95	7.66
**glmed3**	109.95	55.43	7.92
**glmin1**	231.9	16.59	12.6
**glmin2**	261.27	28.31	0
**glmin3**	226.87	54.43	0.97
**iliacus**	480.42	99.35	7.76
**perbrev**	332.44	163.66	14.13
**perlong**	421.04	347.74	9.15
**piri**	677.17	130.94	12.01
**psoas**	545.62	102.55	11.38
**soleus**	2097.6	301.66	17.1
**tfl**	294.91	454.8	2.62
**tibant**	670.1	256.54	10.68
**tibpost**	1156.77	292.57	11.21

Table S8: *Optimized inputs for the lower limb muscle parameters of the K8L subject*

Muscle	MIF [N]	TSL [mm]	PEN [°]
**bflh**	1036.48	339.51	9.54
**bfsh**	261.45	122.83	14.95
**gaslat**	618.44	377.4	7.48
**gasmed**	1480.82	407.77	8.68
**grac**	67	179.88	13.1
**recfem**	1592.04	482.01	15.1
**sart**	70.91	135.66	1.1
**semimem**	806	380.1	14.3
**semiten**	294	259.11	13.73
**vasint**	854.33	204.96	5.23
**vaslat**	1969.91	234.71	12.96
**vasmed**	1223.89	207.05	19.85
**addbrev**	217.66	39.92	11.23
**addlong**	390.33	137.88	14.81
**addmagDist**	460.12	92.06	14.48
**addmagIsch**	365.98	218.31	8.7
**addmagMid**	154.39	47.57	14.02
**addmagProx**	388.21	46.7	18.23
**edl**	87.26	383.24	17.27
**ehl**	67.82	348.44	10.46
**fdl**	146.84	407.5	11
**fhl**	130.75	395.25	15.26
**glmax1**	431.52	53.79	40.24
**glmax2**	693.01	69.9	4.77
**glmax3**	509.38	74.32	42
**glmed1**	342.2	58.19	20.96
**glmed2**	390.32	71.83	31.23
**glmed3**	365.66	49.37	23.08
**glmin1**	193.37	15.65	12.08
**glmin2**	153.76	28.46	0
**glmin3**	158.3	55.33	0.86
**iliacus**	447.99	101.96	16.65
**perbrev**	284.22	150.55	7.73
**perlong**	521.16	341.29	9.97
**piri**	427.17	131.12	9.42
**psoas**	947.72	114.18	9.04
**soleus**	2538.27	263.88	23.13
**tfl**	270.09	482.4	2.79
**tibant**	759.06	252.37	14.89
**tibpost**	877.84	285.55	11.19

References:

1. Ward, S.R., et al., *Are current measurements of lower extremity muscle architecture accurate?* Clin Orthop Relat Res, 2009. **467**(4): p. 1074-82.
2. Lloyd, D.G. and T.F. Besier, *An EMG-driven musculoskeletal model to estimate muscle forces and knee joint moments in vivo.* J Biomech, 2003. **36**(6): p. 765-76.
3. Friederich, J.A. and R.A. Brand, *Muscle fiber architecture in the human lower limb.* J Biomech, 1990. **23**(1): p. 91-5.
4. Fukunaga, T., et al., *Specific tension of human plantar flexors and dorsiflexors.* J Appl Physiol (1985), 1996. **80**(1): p. 158-65.
